# Screening Some Potential Potato Genotypes With an Efficient Photosynthetic System Based on Their Performance Under High Temperature and Irradiance

**DOI:** 10.1155/sci5/5511837

**Published:** 2025-11-29

**Authors:** Muhammad Wasim Haider, Syed Mohsin Abbas, Muhammad Ahmad Saeed, Muhammad Tahir Akram, Tanveer Hussain, Muhammad Nafees, Umar Farooq, Mohammad Valipour, Alina-Stefania Stanciu, Abdulaziz A. Alsahli, Muhammad Bilawal Junaid, Muhammad Waseem, Crossby Osei Tutu

**Affiliations:** ^1^Department of Horticultural Sciences, The Islamia University of Bahawalpur, Bahawalpur 63100, Pakistan; ^2^Department of Horticulture, Faculty of Agricultural Sciences, University of the Punjab, Lahore 54590, Pakistan; ^3^Department of Horticulture, Faculty of Agriculture, PMAS-Arid Agriculture University, Rawalpindi 46300, Pakistan; ^4^Department of Engineering and Engineering Technology, Metropolitan State University of Denver, Denver 80217, Colorado, USA; ^5^Department of Agriculture-Horticulture, Faculty of Environmental Protection, University of Oradea, Oradea 3700, Romania; ^6^Plant Production Department, College of Food and Agriculture, King Saud University, Riyadh 11451, Saudi Arabia; ^7^Department of Food Science and Technology, Faculty of Agriculture and Environment, The Islamia University of Bahawalpur, Bahawalpur 63100, Pakistan; ^8^Department of Family and Consumer Sciences, University of Ghana, Legon, Accra, Ghana

**Keywords:** agronomic and biochemical attributes, chlorophyll fluorescence, genotypic variation, oxidative stress, potato, reactive oxygen species, solar radiation impact

## Abstract

Sustainable potato cultivation in hot, high-light regions such as Southern Punjab, Pakistan, requires identifying genotypes with efficient photosynthetic systems. High temperatures and irradiance often reduce productivity by triggering oxidative stress and limiting photosynthesis. This study evaluated growth, photosynthesis, yield, and biochemical responses in 15 advanced potato genotypes under such conditions. Genotype BD1310-1 showed greater performance with the highest plant height (51.5 cm), leaf area index (1.47), crop growth rate (0.43 g m^−2^ day^−1^), quantum yield of Photosystem II (Φ_II_; 0.75), and tuber yield (21.5 t ha^−1^), along with the lowest oxidative stress indicators. BD1319-2 had the highest number of stems plant^−1^ (5.3), BD1311-4 showed maximum photosynthetically active radiation absorption (430 μmol m^−2^ s^−1^), and BD1335-4 had the highest linear electron flow (209 μmol electrons m^−2^ s^−1^). Principal component analysis grouped traits into positively and negatively correlated clusters. Traits such as LAI, Φ_II_, crop growth rate, and tuber yield were positively associated with photosynthetic efficiency, whereas oxidative stress markers were negatively correlated. The findings suggest that oxidative markers reflect stress, not yield potential. In conclusion, BD1310-1, BD1319-2, BD1311-4, and BD1335-4 demonstrated potential as climate-resilient cultivars suitable for high-temperature (> 40°C) and high-irradiance (> 2000 μmol m^−2^ s^−1^) environments.

## 1. Introduction

Potato is the world's third most consumed and fourth most produced food crop [1–8]. It provides an abundant energy source due to its high carbohydrate and mineral content [9–17]. Pakistan produces 7.94 million tons of potatoes annually from 0.31 million hectares of land, with an average yield of 25.3 tons per hectare [18–21]. The main potato crop in this country is grown in the autumn (October to February). However, market prices are low during harvesting, and fresh potatoes have high values later in the period following the end of March, which can be achieved by cultivating the spring crop (January–May) [22]. The spring crop is hampered by rising temperatures (an increase of 2.7°C from March to May in the last decade) and intense sunlight [23]. A high concentration of salts further aggravates the stress on potato crops [1], particularly during the critical tuber initiation period [24–28]. These ecological stresses impair potato productivity [29–32].

The photosynthetic apparatus, particularly photosystem II (PSII), is more sensitive to high irradiance compared to photosystem I (PSI) [33, 34]. Exposure to high temperatures, particularly above 40°C, can inhibit PSII activity due to protein complex denaturation, impacting overall photosynthetic efficiency and, subsequently, plant health and yield [35–39]. A major physiological consequence of stress is the excessive generation of reactive oxygen species (ROS), including superoxide anion (O^−2^) and hydrogen peroxide (H_2_O_2_). These ROS can cause oxidative damage to lipids, proteins, and nucleic acids, impairing photosynthetic machinery and reducing crop productivity [40–44]. Plants respond to oxidative stress by activating enzymatic antioxidant defenses such as superoxide dismutase (SOD), catalase (CAT), and peroxidases (POD), but the balance between ROS production and scavenging is critical for stress resilience. Therefore, identifying genotypes with an enhanced capacity to maintain efficient PSII function and regulate ROS levels under high light intensity offers a promising strategy for improving potato yield [45, 46]. Assessing the effects of high irradiance on photosynthetic apparatus can provide vital insights into the physiological adaptations of these genotypes [47, 48]. The selection of potato genotypes with stable photosynthetic performance and controlled oxidative stress under intense light can contribute to developing cultivars suited for high-irradiance environments, ultimately enhancing crop productivity and ensuring food security in these regions [49, 50].

To date, various strategies have been implemented to harness the potential benefits presented by environmental challenges [24], including improvement of various cultivation practices such as crop rotation [47, 57], efficient irrigation techniques [52], appropriate nutrient and biostimulant application rates, and frequencies [1, 53, 54], incorporation of organic amendments [55, 56], plant growth promoting organisms [57], integrated pest management [58], and the development of climate-resilient crop varieties [59, 60] to enhance productivity and sustainability. Previous investigations have screened various potato genotypes for overall yield and environmental resilience, but limited research has specifically targeted the photosynthetic efficiency of advanced genotypes under high temperature and irradiance [61]. The heat or light or their combination can cause stress in potato plants, resulting in poor crop growth, development, yield, and quality [62]. Modern breeding techniques for stress tolerance have still failed to identify potato genotypes demonstrating an efficient photosynthetic system during intense light exposure [63, 64]. This gap must be addressed to boost potato production under high irradiance conditions to ensure food security in a changing climate. Potato genotypes must be screened for their high irradiance tolerance in order to address the challenges posed by intense sunlight in Pakistan [61, 65, 66]. Therefore, this study was carried out to identify the advanced genotypes with enhanced PSII systems and evaluate their photosynthetic performance, yield, and endogenous biochemical responses under high irradiance conditions.

## 2. Materials and Methods

### 2.1. Advanced Potato Genotypes

**Figure 1:**
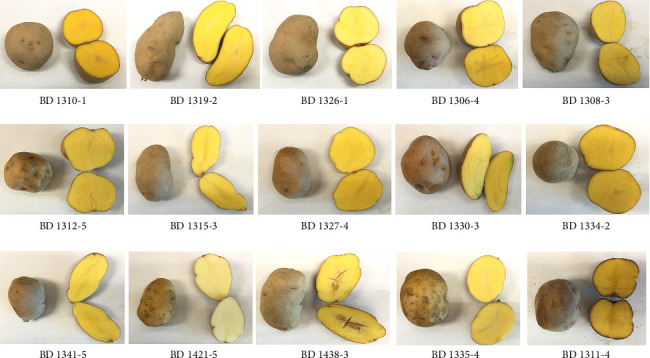
Selected experimental potato genotypes for agronomic, photosynthetic, and biochemical evaluation under high irradiance.

The study comprised the hybrid population *Solanum phureja* × *Solanum stenotomum*, developed for heat tolerance in the United States Department of Agriculture (USDA), including BD1310-1, BD1319-2, BD1326-1, BD1306-4, BD1308-3, BD1312-5, BD1315-3, BD1327-4, BD1330-3, BD1334-2, BD1341-5, BD1421-5, BD1438-3, BD1335-4, and BD1311-4 (Figure 1). The planting material was taxonomically identified and validated by renowned horticulturist Dr. Muhammad Wasim Haider, Lecturer, Department of Horticultural Sciences (DoHS), Faculty of Agriculture and Environment (FA&E), The Islamia University of Bahawalpur (IUB), Pakistan.

### 2.2. Study Area

The experiment was conducted at the Horticulture Experimental Area, DoHS, FA&E, IUB, Pakistan (Latitude 29°22′18.4″ N, Longitude 72°45′57.6″ E, and Altitude 214 m) during two consecutive growing seasons, 2022–23 and 2023–24. The climate in this area is subtropical. The monthly data of air temperature, relative humidity, and rainfall were gathered during the experimental period. The soil in this area is impacted by both salinity and alkalinity. The subsoil water is brackish, with more than 900 mg L^−1^ salts [67]. Before experimenting, the soil samples were taken from 0–15 and 16–30 cm depths of the experimental site by adopting the approach of Ryan [68]. Measurements of soil texture and electrical conductivity [1], pH [69], organic matter [70], phosphorus [71], nitrogen [72], and potassium [73] were made for both layers of soil.

### 2.3. Experimental Procedure

Potato crops were grown in two successive seasons (2022–2023 and 2023–2024), with planting in January and harvesting in April. The healthy, medium-sized tubers (average weight: 60 ± 5 g) were manually planted 15 cm apart and 10 cm deep in ridges made by a tractor-drawn ridge, with a row-row spacing of 75 cm on January 3, 2022. Dehaulming was carried out using “Desica” of Syngenta Private Ltd., and potato tubers were harvested 14 days later. The tubers were harvested on April 28, 2022, during the first year of the experiment. The sowing was carried out on January 5, 2023, and harvesting on April 30, 2023, during the second growing season. Each treatment had a 12.5-m^2^ subplot with a pair of rows. Each row included 54 tubers, for a total of 108 tubers per treatment. The experiment was set up under a randomized complete block design (RCBD) with four replicates per treatment.

### 2.4. Assessment of Attributes

The agronomic, fluorescent, and biochemical assessment of potatoes was carried out from 15 randomly selected potato plants.

### 2.5. Agronomic Attributes

The plant height, number of stems plant^−1^, leaf area index, and crop growth rate were assessed 65 days after sowing. The number of tubers plant^−1^, tuber yield ha^−1^, tuber diameter, and average tuber weight were recorded after harvesting. The leaf area was determined using the formula given below, as previously adopted by Haider et al. [1]. The leaf area index was calculated by dividing the determined leaf area by the ground area covered by the canopy of plants.

(1) $$\begin{array}{c} \mathrm{Log}10\;\left(\text{leaf area in }{\text{cm}}^{2}\right)=2.06\times \mathrm{Log}\;10\;\left(\text{leaf length in cm}\right)-0.458. \end{array}$$

### 2.6. Fluorescent Attributes

The leaf angle, leaf thickness, chlorophyll content, quantum yield of photosystem II, leaf temperature, linear electron flow (LEF), photosynthetically active radiations, total magnitude of electrochromic shift, proton conductivity, proton flux, nonregulatory energy dissipation, and nonphotochemical quenching were recorded from intact potato leaves on the 65th day of sowing from 09:00 a.m. to 11:00 a.m. using a MultispeQ-Beta instrument and PhotosynQ platform software [74].

### 2.7. Biochemical Attributes

The measurement of O^−2^ in the potato leaf tissues was carried out using the method described by Hasan et al. [75]. The H_2_O_2_ content was calculated using the approach adopted by Haider et al. [76]. To determine the antioxidative enzyme content, the mature leaves of potato plants were crushed using a cold mortar and pestle and homogenized with 2 mL of phosphate buffer (pH = 7). After that, the mixture was centrifuged for 5 min at 4°C at 10,000 rpm in a Rotofix 46 centrifuge (Hettich, Kirchlengern, Germany). Then, the antioxidative enzyme concentration was measured after the collection of the supernatant. The activities of SOD (EC 1.15.1.1), CAT (EC 1.11.1.6), ascorbate POD (APX) (EC 1.11.1.11), and POD (EC 1.11.1.7) were calculated according to the procedure used by Haider et al. [76].

### 2.8. Statistics

The obtained data were arranged using Microsoft Excel 2016 and subjected to ANOVA using Statistix 9 for Windows (Analytical Software, Tallahassee, USA). The mean values were compared with the least significant difference (LSD) test at *p* ≤ 0.05. The principal component analysis was performed using XLSTAT 2022.1.

## 3. Results

### 3.1. Air Temperature, Humidity, Rainfall, and Sunshine Trend

**Figure 2:**
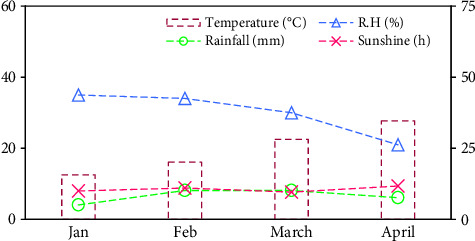
The average values of air temperature, rainfall, relative humidity, and sunshine were noted over the course of the study during 2022-23 and 2023-24 at the experimental site.

The mean air temperature showed a gradually increasing trend from January (12.5°C) to April (27.7°C) (Figure 2). Alternatively, the relative humidity declined from the first month (January) (35%) to the last month (April) (21%) of the experimental period during both growing seasons (Figure 2). Rainfall increased from January (5.08 mm) to February (10.2 mm) (Figure 2). However, it remained constant until March (Figure 2). Then, a substantial reduction (25.5%) was noticed in the rainfall during April (7.6 mm) (Figure 2). A noticeable increase in the sunshine period was observed from January (10 h) to February (11 h) (Figure 2). Nevertheless, it was reduced in March (9.6 h) (Figure 2). April had the highest (11.7 h) period of sunshine (Figure 2).

### 3.2. Analysis of the Soil and Irrigation Water's Physicochemical Properties

The physicochemical properties of the above 0–15 cm and 16–30 cm layers of soil are displayed in Table 1. The soil was alkaline, with pH varying between 8.0 and 8.3 and EC ranging from 1.55 to 1.72 dS m^−1^ (Table 1). The soil had sand particles ranging from 55% to 58%, clay particles from 14% to 19%, and silt particles from 26% to 28% (Table 1). Therefore, the textural class was designated as sandy loam (Table 1). The mean values of organic matter in the soil ranged from 0.75% to 0.81% (Table 1). The average soil cation exchange capacity (CEC) ranged from 7.0 to 7.7 c mol kg^−1^ (Table 1). This could be due to greater quantities of salts (0.65–0.79 mg g^−1^) in the soils of the study area (Table 1). The average nitrogen concentration in the soil ranged from 0.040% to 0.051% (Table 1). The mean values of phosphorus and potassium in the soil ranged from 8.5 to 11.0 and 124 to 150 mg kg^−1^, respectively (Table 1). The physicochemical properties of irrigation water are displayed in Table 2. There was a moderate risk of alkalinity and sodicity in the irrigated water (Table 2).

### 3.3. Agronomic Attributes

**Figure 3:**
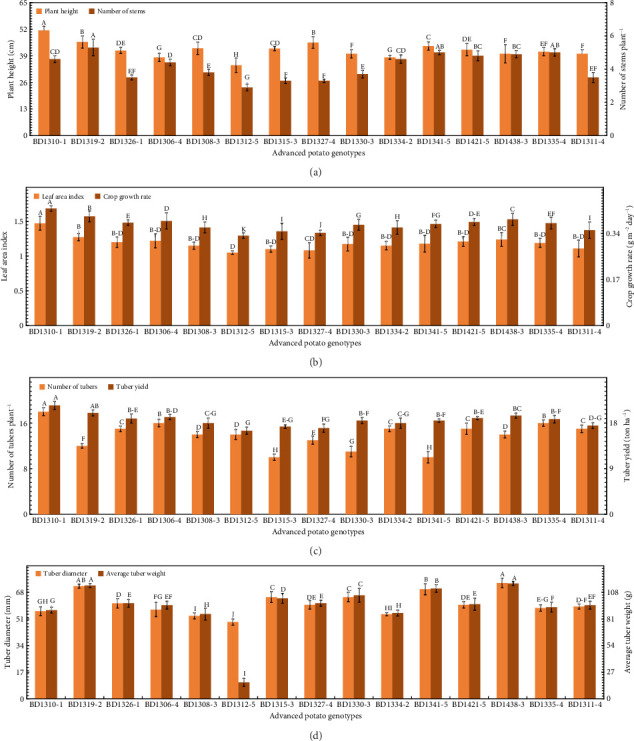
Agronomic attributes including plant height and number of stems plant^−1^ (a), leaf area index and crop growth rate (b), number of tubers plant^−1^ and tuber yield (c), and tuber diameter and average tuber weight (d) of 15 advanced potato genotypes under high irradiance. The vertical bars (±) denote the mean values of standard error. The letters above the vertical bars represent the variations between treatment means based on the LSD test at *p* ≤ 0.05 following analysis of variance (ANOVA).

Agronomic attributes are critical to determining the performance of potato genotypes in response to high temperature and light stress. In the present study, the greatest plant height (51.5 cm) was observed in BD1310-1, whereas the number of stems plant^−1^ (5.3) in BD1319-2, statistically at par with BD1341-5 (5.0) and BD1335-4 (5.0) (Figure 3(a)). The smallest plant height (34.4 cm) and number of stems plant^−1^ (2.9) were noticed in BD1312-5 (Figure 3(a)). The highest leaf area index (1.47) and crop growth rate (0.43 g m^−2^ day^−1^) were noted in BD1310-1 (Figure 3(b)). The lowest leaf area index (1.05) was noted in BD1312-5, statistically comparable to BD1327-4 (1.08), BD1311-4 (1.11), BD1334-2 (1.15), BD1330-3 (1.17), BD1341-5 (1.18), BD1335-4 (1.19), and BD1421-5 (1.21) (Figure 3(b)). The lowest crop growth rate (0.33 g m^−2^ day^−1^) was observed in BD1312-5 (Figure 3(b)). Similarly, the highest number of tubers plant^−1^ (18) was recorded in BD1310-1 (Figure 3(c)). The highest tuber yield was found in BD1310-1 (21.5 tons ha^−1^), statistically at par with BD1319-2 (20 tons ha^−1^) (Figure 3(c)). The lowest number of tubers plant^−1^ (10) was found in BD1315-3 (Figure 3(c)). The lowest tuber yield (16.5 tons ha^−1^) was found in BD1312-5, statistically comparable with BD1311-4 (17.5 tons ha^−1^), BD1327-4 (17 tons ha^−1^), BD1308-3 (18 tons ha^−1^), and BD1334-2 (18 tons ha^−1^) (Figure 3(c)). The highest tuber diameter (74 mm) was recorded in BD1438-3, statistically at par with BD1319-2 (72 mm) (Figure 3(d)). The highest average tuber weight (117 g) was noted in BD1438-3, significantly similar to BD1319-2 (115 g) (Figure 3(d)). The lowest tuber diameter (49 mm) and average tuber weight (117 g) were noted in BD1312-5 (Figure 3(d)).

### 3.4. Fluorescent Attributes

**Figure 4:**
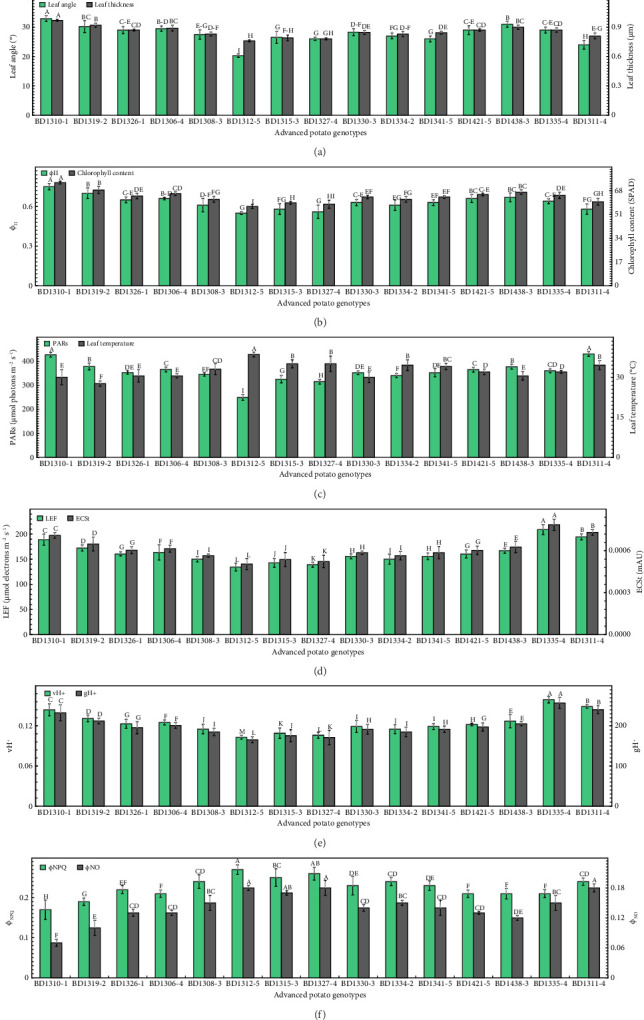
Fluorescent attributes including leaf angle and leaf thickness (a), quantum yield of Photosystem II (ϕ_II_) and chlorophyll content (b), photosynthetically active radiations (PARs) and leaf temperature (c), linear electron flow (LEF) and total magnitude of electrochromic shift (ECSt) (d), proton flux (vH^+^) and proton conductivity (gH^+^) (e), and nonphotochemical quenching (ϕ_NPQ_) and nonregulatory energy dissipation (ϕ_NO_) (f) of 15 advanced potato genotypes under high irradiance. The vertical bars (±) denote the mean values of standard error. The letters above the vertical bars represent the variations between treatment means based on the LSD test at *p* ≤ 0.05 following analysis of variance (ANOVA).

Fluorescence-related attributes are essential for evaluating the efficiency of the photosynthetic apparatus of potato genotypes in response to high temperature and light stress. In this study, the highest leaf angle (32.9°) and leaf thickness (0.97 μm) were recorded in BD1310-1 (Figure 4(a)). The lowest leaf angle (20.3°) was recorded in BD1312-5 (Figure 4(a)). The lowest leaf thickness (0.76 μm) was recorded in BD1312-5, statistically comparable to BD1327-4 (0.78 μm) and BD1315-3 (0.79 μm) (Figure 4(a)). The greatest quantum yields of photosystem II (ϕ_II_) (0.75) and chlorophyll content (73.8) were found in BD1310-1 (Figure 4(b)). The smallest values (0.55) for ϕ_II_ were noted in BD1312-5, significantly similar to BD1327-4 (0.56), while comparable to BD1315-3 (0.58), BD1311-4 (0.58), and BD1334-2 (0.61) (Figure 4(b)). The lowest chlorophyll content (56.7) was noted in BD1312-5, although not significantly different from BD1327-4 (58.4) (Figure 4(b)). The greatest photosynthetically active radiations (PARs) (430 μmol photons m^−2^ s^−1^) were recorded in BD1311-4, significantly similar to BD1310-1 (426.5 μmol photons m^−2^ s^−1^) (Figure 4(c)). The smallest PARs (249 μmol photons m^−2^ s^−1^) were recorded in BD1312-5 (Figure 4(c)). Alternatively, the highest leaf temperature (38.5°C) was noted in BD1312-5 and the lowest leaf temperature (27.6°C) in BD1319-2 (Figure 4(c)). The strongest LEF (209.1 μmol electrons m^−2^ s^−1^) and magnitude of electrochromic shift (ECSt) (786 × 10^−6^) were noticed in BD1335-4 (Figure 4(d)). The weakest LEF (142.7 μmol electrons m^−2^ s^−1^) and ECSt (505 × 10^−6^) were noticed in BD1312-5 (Figure 4(d)). The values of proton flux (vH^+^) (0.159) and proton conductivity (gH^+^) (257.2) were highest in BD1335-4 (Figure 4(e)). The lowest values of vH^+^ (0.103) and gH^+^ (165.32) were found in BD 1312-5 (Figure 4(e)). The highest nonphotochemical quenching (ϕ_NPQ_) (0.27) was noted in BD1312-5, statistically comparable to BD1327-4 (0.26) (Figure 4(f)). The highest nonregulatory energy dissipation (ϕ_NO_) (0.18) was recorded in BD1312-5 and BD1311-4 (Figure 4(f)). The lowest ϕ_NPQ_ (0.17) and ϕ_NO_ (0.07) were observed in BD1310-1 (Figure 4(f)).

### 3.5. Biochemical Attributes

**Figure 5:**
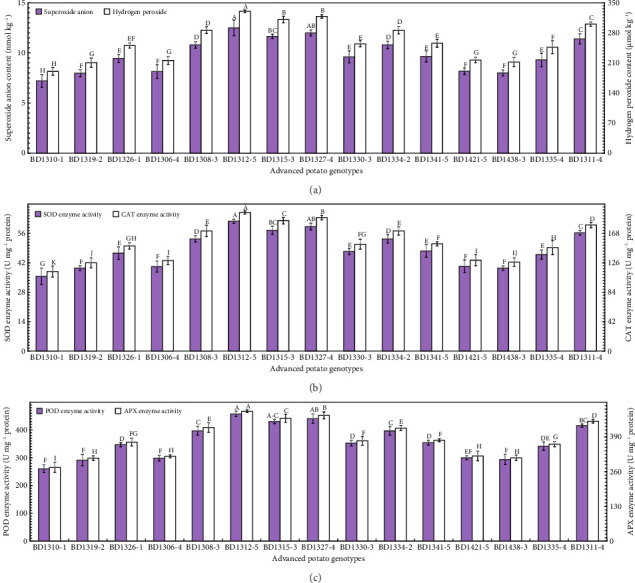
Biochemical profiling including superoxide anion and hydrogen peroxide content (a), superoxide dismutase (SOD) and catalase (CAT) enzyme activities (b), and peroxidase (POD) and ascorbate peroxidase (APX) enzyme activities (c) in the leaves of 15 advanced potato genotypes under high irradiance. The vertical bars (±) denote the mean values of standard error. The letters above the vertical bars represent the variations between treatment means based on the LSD test at *p* ≤ 0.05 following analysis of variance (ANOVA).

Biochemical attributes are critical for evaluating oxidative stress tolerance in advanced potato genotypes to high temperature and irradiance, aiding in the selection of genotypes with enhanced resilience and performance. In the current study, the highest superoxide anion (O^−2^) content (12.5 nmol kg^−1^) was noted in BD1312-5, statistically at par with BD1327-4 (12.0 nmol kg^−1^) (Figure 5(a)). The highest hydrogen peroxide (H_2_O_2_) content (330.5 μmol kg^−1^) was noted in BD1312-5 (Figure 5(b)). The lowest O^−2^ was noted in BD1310-1 (7.2 nmol kg^−1^) and H_2_O_2_ in BD1319-2 (210.5 μmol kg^−1^) (Figure 5(a)). The highest SOD enzyme activity was noted in BD1312-5 (61.8 U mg^−1^ protein), statistically comparable to BD1327-4 (59.3 U mg^−1^ protein) (Figure 5(b)). The highest CAT enzyme activity (197.9 U mg^−1^ protein) was noted in BD1312-5 (Figure 5(b)). The lowest SOD (35.6 U mg^−1^ protein) and CAT (113.5 U mg^−1^ protein) enzyme activities were noted in BD1310-1 (Figure 5(b)). The highest POD enzyme activity (457.5 U mg^−1^ protein) was noted in BD1312-5, statistically comparable to BD1327-4 (440.5 U mg^−1^ protein) and BD1315-3 (430.4 U mg^−1^ protein) (Figure 5(c)). The lowest POD enzyme activity was noted in BD1310-1 (260.3 U mg^−1^ protein), statistically similar to BD1319-2(291.5 U mg^−1^ protein), BD1438-3 (293.8 U mg^−1^ protein), and BD1306-4 (298.8 U mg^−1^ protein), and statistically comparable to BD1421-5 (300 U mg^−1^ protein) (Figure 5(c)).

### 3.6. Principal Component Analysis

**Figure 6:**
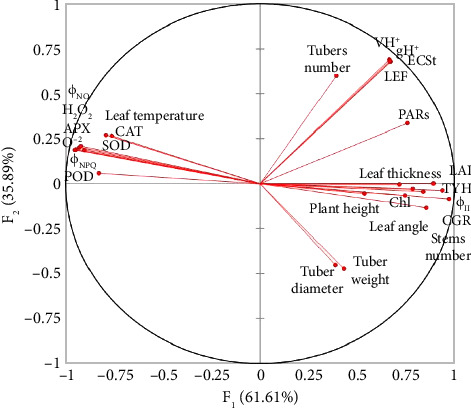
The cluster of studied attributes into positively and negatively correlated groups. LAI = leaf area index, CGR = crop growth rate, TYH = tuber yield per hectare, ϕ_II_ = quantum yield of Photosystem II, Chl = chlorophyll content, PARs = photosynthetically active radiations, LEF = linear electron flow, ECSt = total magnitude of electrochromic shift, vH^+^ = proton flux, gH^+^ = proton conductivity, ϕ_NPQ_ = nonphotochemical quenching, ϕ_NO_ = nonregulatory energy dissipation, O^2^ = superoxide anion, H_2_O_2_ = hydrogen peroxide, CAT = catalase, SOD = superoxide dismutase, POD = peroxidase, and APX = ascorbate peroxidase.

The biplot illustrates the clustering of various agronomic, fluorescent, and biochemical attributes of potato genotypes under high temperature and irradiance conditions (Figure 6). The studied attributes are grouped into positively and negatively correlated clusters along two principal components, F_1_ (61.61% variance) and F_2_ (35.89% variance), accounting for a combined 97.5% of the data variation (Figure 6).

The attributes located on the right side of the plot (e.g., number of tubers plant^−1^, average tuber weight, leaf thickness, leaf angle, leaf area index, and chlorophyll fluorescence attributes) are positively correlated with each other and with the primary axis (F_1_) (Figure 6). These attributes are associated with enhanced photosynthetic efficiency and productivity under high temperature and irradiance, indicating that genotypes excelling in these attributes likely exhibit enhanced performance in terms of growth and yield in stress conditions (Figure 6).

In contrast, the attributes on the left side of the plot like O^−2^ and H_2_O_2_ content, and SOD, CAT, and POD enzyme activities, are biochemical attributes of oxidative stress and ROS scavenging (Figure 6). This grouping suggests that these stress-related attributes are inversely correlated with the attributes contributing to productivity, as indicated by their negative association with F_1_ (Figure 6). This inverse relationship underscores that genotypes with efficient antioxidant mechanisms may exhibit resilience under high-temperature and irradiance conditions but might not necessarily translate these into higher yield-related traits (Figure 6).

## 4. Discussion

**Figure 7:**
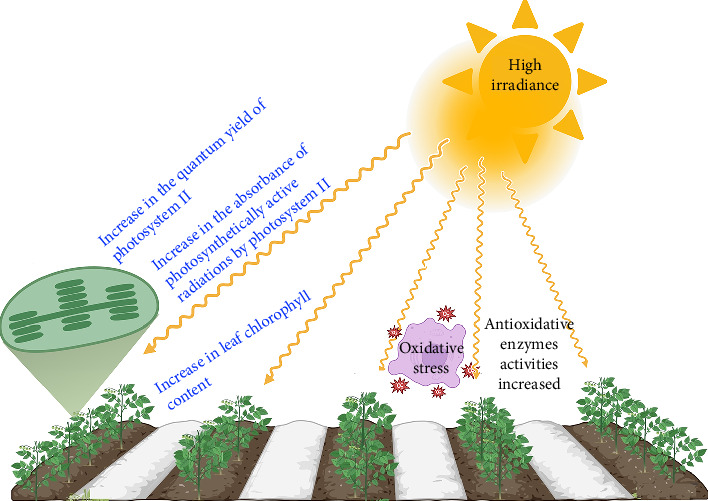
A visual illustration of the mechanism of an efficient photosynthetic system in potato under high irradiance.

The present 2-year study focuses on shortlisting some advanced potato genotypes based on their adaptability and performance under high temperature and irradiance, aiming to pinpoint candidates with optimized photosynthetic systems that can thrive in regions with high temperature (40°C) and intense sunlight (2000 μmol m^−2^ s^−1^). High temperature during the cropping season (Figure 2), particularly at tuber initiation stage, which initiates within the 50 days after sowing [35], can impair photosystem II (PSII) function and reduce chlorophyll content, leading to lower fluorescence yield and reduced photosynthetic efficiency [36–38, 77]. The potato genotypes underwent high-temperature conditions throughout both growing seasons (2022–24), particularly during tuber initiation and tuber bulking. The average plant height, number of stems plant^−1^, leaf area index, crop growth rate, number of tubers plant^−1^, tuber yield ha^−1^, tuber diameter, and average tuber weight were found significantly different among the studied genotypes (Figure 3). The results are consistent with those of Rykaczewska [78] and Demirel et al. [79], who reported substantial variations among potato genotypes under high temperatures. In another study, Kleinwechter et al. [49] found a wide cultivar variation for growth and yield attributes under contrasting environments, which is beneficial for future climate change. Similarly, Paul et al. [80] found significant differences between potato cultivars for carbon assimilation, antioxidant enzyme activities, and tuber yield. Furthermore, in a field study by Angmo et al. [81], potato genotypes exhibited a significant variability for morpho-anatomical attributes under frost stress. The improved vegetative and tuber growth of BD1310-1 and BD1319-2 under high temperature and irradiance might be due to their efficient photosynthetic and antioxidant defense system (Figure 7).

The thickness of the leaf had a strong positive correlation with the number of stomata mm^−2^, subcellular CO_2_, photosynthetic rate, and Φ_II_ in previous studies [82–86]. The genotypes with thick leaves, including BD1310-1 (Figure 4(a)), might have a higher concentration of chloroplasts, enabling the plants to absorb more light and improving subcellular CO_2_ transport within the leaves, which in turn increased the photosynthetic efficacy of potato plants. The thickness of leaves is beneficial for photosynthesis because chloroplasts, even those positioned deeper within the leaves, can absorb a sufficient amount of light [87]. On the other hand, leaf thickness showed a strong negative connection with transpiration rate and stomatal conductance in earlier investigations [88], which are associated with water transfer. This study also found a positive correlation between leaf area index and leaf thickness with Φ_II_, which indicates that potato plants with thick leaves may have a high number of chloroplasts per unit leaf area (Figure 6). The chlorophyll fluorescence is a measurement of the amount of light energy captured by chlorophyll molecules in plants and reemitted as fluorescence light [89, 90]. It reveals useful information about the photosynthetic capability of plants [91]. The chlorophyll fluorescence occurs in chloroplasts, which are responsible for converting light energy into chemical energy in plants [92]. Thus, chlorophyll fluorescence measurements are an important technique for assessing the photosynthetic apparatus of germplasm and comprehending their physiological consequences on chloroplasts. In the current research, leaf angle was measured due to its substantial impact on chlorophyll fluorescence by regulating light absorption, distribution, and physiological responses to varied light circumstances [93]. The largest quantity of light is received by the leaves, which are oriented perpendicular to the incoming sunlight. This enables chloroplasts to efficiently commence photosynthesis by absorbing more photons. On the other hand, obliquely orientated leaves receive less direct light, which results in lower rates of photosynthesis and less chlorophyll excitation [94]. The striking PARs are another key photosynthetic parameter, representing the proportion of 400–700 nm wavelength light that can be used for photosynthesis. In the current study, leaf angle had a strong positive correlation with PARs (Figure 6), indicating that leaves inclined broadly toward incident light received more PARs, hence improving photosynthesis. The results are consistent with those of Haider et al. [95], who assessed the transmittance rate of PARs in spinach, and Yirdaw and Luukkanen [96], who measured the transmittance rate of PARs in five distinct plant species and discovered that leaf angle plays a critical role in light absorption for photosynthesis.

ROS, including superoxide anion (O^−2)^ and hydrogen peroxide (H_2_O_2_), are commonly overproduced in plants under abiotic stress conditions such as salinity, drought, high temperature, and intense light [97]. These reactive molecules, if not adequately detoxified, can damage cellular components including lipids, proteins, and nucleic acids, leading to oxidative stress and impaired physiological function [98]. In this study, considerable genotypic variation was observed in ROS levels, with some genotypes maintaining lower O^−2^ and H_2_O_2_ accumulation. The enzyme activities of SOD, CAT, POD, and ascorbate peroxidase (APX) were also found to vary among genotypes which function collectively to scavenge ROS and protect cellular structures [99]. Genotypes with moderate ROS levels and balanced antioxidative enzyme activities demonstrated improved photosynthetic efficiency and less photo-oxidative damage. Notably, ROS levels showed a strong positive correlation (*r ≥ 0.78*) with SOD, CAT, POD, and APX, indicating that higher ROS production stimulated greater antioxidant responses. Conversely, genotypes with inherently lower ROS accumulation exhibited reduced enzyme activities, reflecting a lower oxidative burden. In this study, the genotypes BD1310-1, BD1319-2, and BD1335-4 with enhanced heat and irradiance tolerance showed a lower ROS generation and hence lower antioxidative enzyme activities including SOD, CAT, APX, and POD (Figure 5). These results suggest that the ability to regulate ROS metabolism is an important trait contributing to heat and irradiance tolerance in potato, aligning with findings from Li et al. [37] and Paul et al. [80]. Our study has successfully identified advanced potato genotypes exhibiting enhanced photosynthetic efficacy under high temperature and irradiance conditions. Their selection will provide a foundation for future breeding programs aimed at developing climate-resilient potato varieties.

## 5. Conclusion

In conclusion, the study identified BD1310-1, BD1319-2, BD1311-4, and BD1335-4 as the most promising genotypes under high-temperature and irradiance conditions due to their higher photosynthetic efficiency, growth, and tuber yield. The findings emphasize the potential of using specific fluorescent and biochemical attributes to screen the genotypes for resilience and productivity in stress-prone environments, providing valuable insights for breeding programs in hot regions.

## Figures and Tables

**Table 1:** Analysis of the soil's physicochemical properties during 2022-23 and 2023-24.

Particular	Mean values obtained for both soil layers		Unit
	0–15 cm	16–30 cm	
Silt	26	28	%
Sand	55	58	%
Clay	19	14	%
Soil texture	Sandy loam	Sandy loam	—
Total dissolved salts	0.65	0.79	mg g^−1^
pH	8.0	8.3	—
EC	1.55	1.72	dS m^−1^
Saturation	34	31	%
Organic matter	0.81	0.75	%
CEC	7.7	7.0	c mol kg^−1^
Phosphorus content	8.5	11.0	mg kg^−1^
Nitrogen content	0.051	0.040	%
Potassium content	150	124	mg kg^−1^

**Table 2:** Analysis of the physicochemical properties of the irrigated water during 2022-23 and 2023-24.

Particular	Mean value	Unit
Total dissolved salts	1.12	g L^−1^
Ca^+2^ + Mg^+2^	7.12	Meq L^−1^
Na^+1^	4.70	Meq L^−1^
HCO_3_^−1^	2.46	Meq L^−1^
Cl^−1^	1.05	Meq L^−1^
CO_3_^−2^	—	Meq L^−1^
EC	1.18	dS m^−1^
pH	7.7	—
Sodium adsorption ratio	2.41	—
Residual sodium carbonate	0.30	Meq L^−1^

## Data Availability

The data used to support the findings of this study are included within the article.
